# A Novel Growing Device Inspired by Plant Root Soil Penetration Behaviors

**DOI:** 10.1371/journal.pone.0090139

**Published:** 2014-02-25

**Authors:** Ali Sadeghi, Alice Tonazzini, Liyana Popova, Barbara Mazzolai

**Affiliations:** 1 Center for Micro-BioRobotics, Istituto Italiano di Tecnologia, Pontedera, Italy; 2 The BioRobotics Institute, Scuola Superiore Sant’Anna, Pontedera, Italy; University of Vermont, United States of America

## Abstract

Moving in an unstructured environment such as soil requires approaches that are constrained by the physics of this complex medium and can ensure energy efficiency and minimize friction while exploring and searching. Among living organisms, plants are the most efficient at soil exploration, and their roots show remarkable abilities that can be exploited in artificial systems. Energy efficiency and friction reduction are assured by a growth process wherein new cells are added at the root apex by mitosis while mature cells of the root remain stationary and in contact with the soil. We propose a new concept of root-like growing robots that is inspired by these plant root features. The device penetrates soil and develops its own structure using an additive layering technique: each layer of new material is deposited adjacent to the tip of the device. This deposition produces both a motive force at the tip and a hollow tubular structure that extends to the surface of the soil and is strongly anchored to the soil. The addition of material at the tip area facilitates soil penetration by omitting peripheral friction and thus decreasing the energy consumption down to 70% comparing with penetration by pushing into the soil from the base of the penetration system. The tubular structure provides a path for delivering materials and energy to the tip of the system and for collecting information for exploratory tasks.

## Introduction

Soil exploration mechanisms are among the most investigated systems because of their extremely wide range of applications. Soil is a source of vital elements (water, nutrients, and minerals) for all living systems, containing the major energy resources used by humanity and providing important elements that enable technological advancement. As a consequence of this exploitation, the effects of human activity on soil are extremely serious (soil features and functions can be recovered only after a long time and with high costs), and for this reason, long-term monitoring is needed, with particular attention to physical soil properties, the presence of contaminants (i.e., heavy metals and organic pollutants) and nutrients (i.e., phosphorous and nitrogen), and water conditions in shallow depths. The techniques used in soil monitoring commonly involve sensorized probes, which are pushed into the soil to certain depths. A multitude of developed mechanical penetration systems, most of which are based on electrical, pneumatic, and hydraulic actuators [1]–[7], have been developed, both for taking samples and for creating access for sensorized probes, which need to be introduced directly at a certain depth [8]. Among these systems, rotary drilling is the most widely used. Drilling devices are able to create straight and vertical boreholes in various substrates (e.g., sand, rock, and ice) [5]–[7]: the drill bit, which is typically actuated by an electric motor fixed on the body of the device, penetrates the medium perpendicularly to the drill axis, being forced against the bottom of the bore by the weight of the entire drill string. The required weight on the bit may be significant (depending on the type of soil), and in several operating conditions, the exploitable load is limited due to friction, buckling or a reduced vertical depth [9]. Moreover, drilling methods can produce local heating due to the cutting/shearing process and need lubricants or fluids to remove soil particles or dirty [10], these representing limiting factors when the aim is to find life or water signatures (e.g., in space missions) [2], [11]. The most common alternative to drilling and sampling methods for soil testing in agricultural [12], [13] and geotechnical practice [14], [15] involves the use of sensorized steel probes (usually with a cone or a blunt tip) called ‘penetrometers,’ which are pushed from the top of the devices into the soil manually or by actuators on the soil surface. In agriculture, penetrometers are used at relatively shallow depths for characterizing soil strength properties (in particular, penetration resistance to root growth), which change under the influence of climate, plant growth, and soil management and which largely depend on bulk density, moisture content, and soil texture [16], [17]. As previously reported [18], 40–80% of the measured soil resistance to a metal probe is frictional. To reduce this frictional component, which increases the penetrometer resistance to between 2 and 8 times that of the real root penetration resistance, penetrometers with a lubricated tip and with a rotating tip have been developed [19]. In geology and civil engineering, push rods are needed to achieve deep layers in field testing: the rods are inserted by a thrust mechanism counterbalanced by a reaction system (pushing equipment) [20]. The thrust capacity needed varies depending on the type of soil, and its maximum capacity is limited not only by the thrust mechanism performance but also by damage to the push rods, including buckling [14].

In this context, robotic devices could assist in identifying potentially useful or hazardous areas and in monitoring levels of essential elements or geological properties. However, robotic technology for soil exploration and monitoring has been scarcely developed compared with that which is available for exploration and monitoring above ground and underwater. This situation is partly a consequence of the physical constraints of underground operation, which make the task extremely challenging for an autonomous system [2]. The requirement for high dexterity in the exploration of underground unstructured environments has led to a growing interest in biologically inspired solutions [21], [22]. Various penetration devices have been developed that mimic particular aspects of animals such as wood wasps, locusts, and clam [23]–[25]. These previous works were mainly focused on burrowing and drilling animals, which penetrate substrates by exploiting the movements produced by their muscular systems; but plants are the most efficient at soil exploration among living organisms, being able to grow and adapt inside the medium. They develop networks of growing and branching roots whose tips (apices) are highly sensitive. Plants efficiently explore the soil for minerals and water to fulfill their primary functions in a wide variety of environments. Each root apex is able to sense a variety of chemical, physical, and mechanical stimuli [26]–[28]. This information is integrated within genetically driven developmental rules and shared with the entire network of roots to improve plant fitness [29]–[31].

Unlike animals, for which growth is related to development, the distinctive feature of roots is the link between their growth and their movement [32]. Plant roots are able to find low-resistance pathways and exploit cracks in the soil, overcoming soil penetration resistance [33]–[35]. Their exploration capability arises from the root apex [36]–[38]; i.e., the plant root moves and penetrates soil by growing at the apical region [39]–[40]. The growth process in the tip enables the roots to adapt their morphology and organ development to the environmental conditions such as the soil texture and mechanical impedance because the cell division and morphology are directly influenced by the interaction with the surrounding environment [41]–[43]. In addition, the apex morphology has been demonstrated to have a significant role in soil penetration [44]–[46].

**Figure 1:**
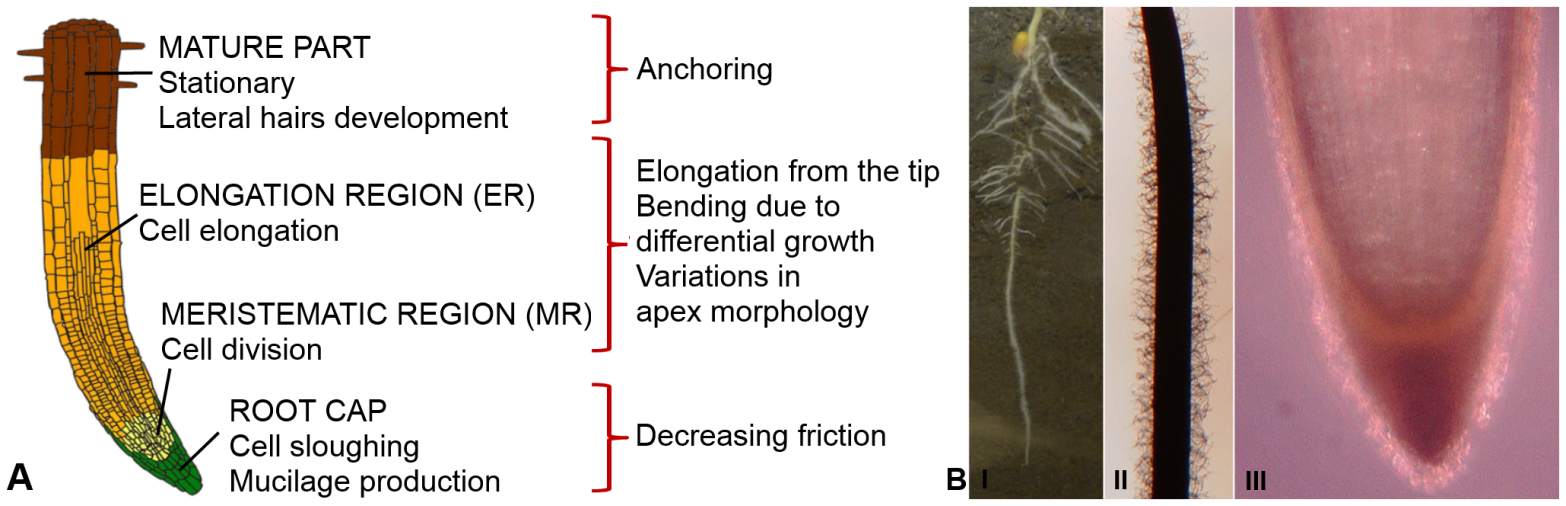
Plant root structures and functions. Figure (a) illustrates plant root structures and their functions. Four main regions can be identified, each with different roles. New cells are created by mitosis in the meristematic region (MR). These cells are then elongated by osmotic pressure and move to the elongation region (ER). Cell division and cell elongation provide the force to penetrate into the soil; asymmetries with respect to the root axis results in bending. The mature root consists of elongated cells that are stationary and provide a strong anchor to the soil, thus supporting tip penetration. Root cap cells exude mucilage and slough off, which decreases friction during penetration by lubricating the surrounding soil. Figure (b) shows images of an actual maize (*Zea mays*) root; the images were captured with a digital microscope (HIROX KH-7700). Specifically, image I is a two-week old maize root growing in soil, in which the primary root, the lateral roots and several seminal roots can be observed. Image II shows the mature region of the primary root with visible root hairs. Image III shows the apex of the primary root with a distinguishable root cap. Image IV shows the root cap of the primary root with sloughed cells visible around it.

**Figure 2:**
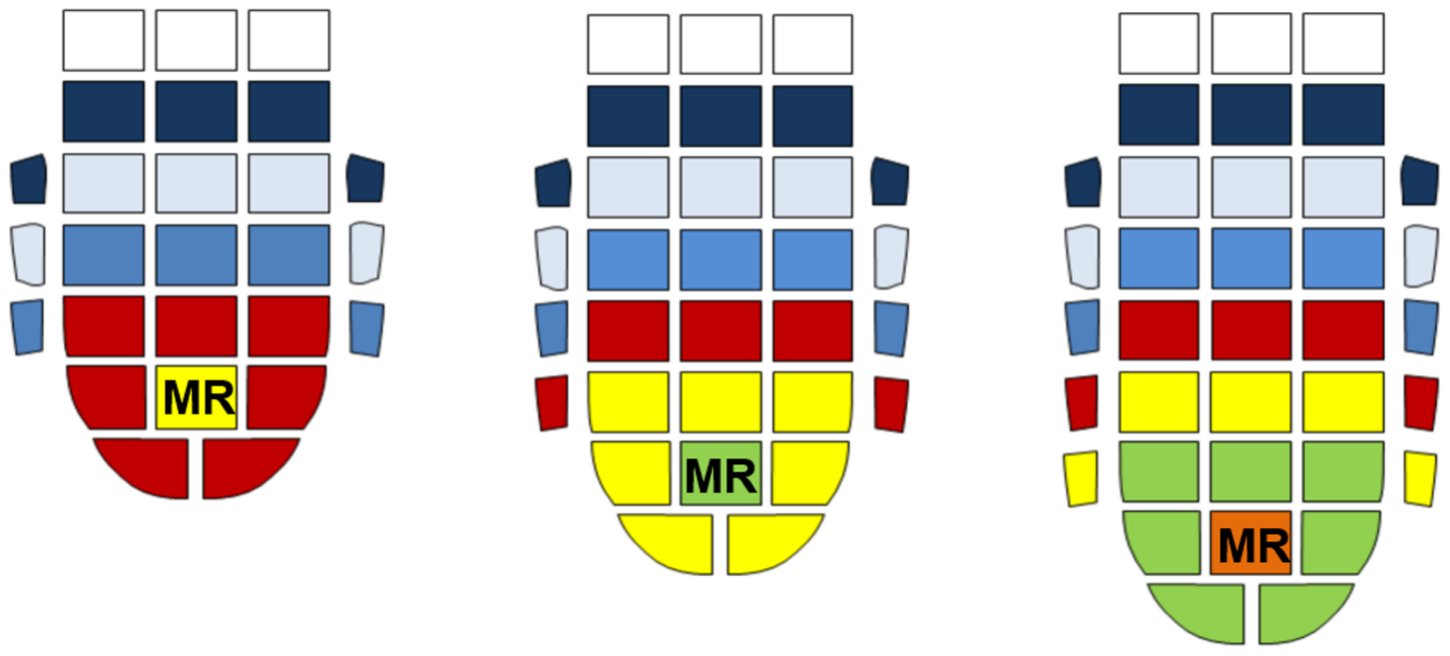
Elongation from the tip (EFT) process during three following steps of growth. The colors indicate the movement of cells from the MR to the mature root and sloughing; mature cells do not move with respect to the soil, whereas the apex is forced into the soil by the new cells originating in MR.

More specifically, root development is driven by two continuous processes in the apex: cell division and cell elongation, which occur in the meristematic and elongation regions (Fig. 1a, b), respectively. In this work, we term this growth process “elongation from the tip” (EFT) (Fig. 2, Video S1). Newly generated cells move from the meristematic region to the elongation region, where they expand axially because of the water absorbed by osmosis and the directional loosening of the cell wall [47], [48]. This action allows the root to penetrate the soil with only a small part of its structure (the tip), while the remainder of the structure is stationary and in contact with the soil (the mature part, shown in Fig. 1a, b). This process provides the pressure required for the forward advancement of the root. The exerted pressure, up to 1 MPa, is dissipated in the expansion of a cavity in the soil and in the frictional resistance of the soil to the advancement of the root [49]–[51]. The penetration may be straight or curved, depending on whether the cell growth is symmetric or differential. Cap cells are continually produced in the meristematic region. These cells move to the root cap and then slough off from its outer surface while producing mucus [52]. In this way, the cap cells create an interface between the soil and the root apex (Video S1). The root cap protects the delicate cells in the meristematic region, and the mucus promotes root penetration by reducing friction [52]–[55]. Mature cells, which are situated behind the apex, are strongly anchored to the soil and thus allow the apex to move forward. This anchorage, also called root-soil adhesion [56], is achieved by root hairs, secondary roots, and the root architecture (Fig. 1b) [56]–[58]. Stronger root-soil adhesion may enable the root to penetrate harder soils [59].

The mechanical impedance of the soil directly influences the root growth rate and shapes the apex morphology and the overall root architecture [60], [61]. Generally, when a root encounters a higher mechanical impedance, the elongation rate decreases and the apex diameter increases [12], [62]. A larger root radius reduces the axial resistance of the soil immediately in front of the root and provides a stronger anchorage behind the penetrating apex [63], [64].

A plethora of studies on the growth kinematics and the morphological features of plant roots have been performed to understand root penetration behaviors [49], [57], [65]. These studies mainly involved observations of living roots in various environmental conditions [66] and computational modeling and simulation of root movements [63], [67], [68]. A recent trend in this field is the development of new tools for studying root kinematics that follow the path taken over time by a recognizable part of the observed root system, which may lead to the discovery of new features of the mechanism of root growth [42], [43], [69], [70], [71]. Taking an engineering approach to the study of plant roots allows important principles to be extracted and used for designing innovative exploratory robots. In [72], the development of a self-anchoring system for soil penetration that was inspired by the sloughing cells in the apical root region described previously was reported. Tools for validating plant features were presented and the efficiency of this biological phenomenon was demonstrated quantitatively. In addition, the necessity of anchorage for successful substrate penetration was discussed. A quantitative evaluation of the role of EFT in decreasing the energy consumption in soil penetration was recently performed and described in [46]. That study demonstrated how penetration by EFT reduces dynamic friction and energy consumption in the root. Two sets of penetration tests were performed in granular substrates using a purposely built probe [46] to compare the performance of EFT with that of penetration by applying a downward force at the top of the probe (identified as “no elongation from the tip”, NoEFT). These tests demonstrated that the penetration energy required for EFT was less than that for NoEFT in soil penetration tasks at various initial depths; the reduction increased from approximately 20% at an initial depth of 100 mm to 50% at an initial depth of 250 mm. The trend of these results shows that at greater depths, a significant reduction in energy consumption (more than 50%) can be obtained with the EFT approach. Therefore, EFT in a plant root represents an efficient solution for soil penetration, and the preliminary results reported in [46] were considered a starting point and a source of inspiration for designing and developing an innovative robotic soil exploration system. In this paper, we present a new generation of devices that are able to grow and construct their own body and were inspired by the growth and soil penetration behaviors in plant roots. The proposed root-like device grows through a monotonic process that continuously adds new material. This device can efficiently penetrate soil by taking advantage of root penetration behaviors, in particular EFT. However, because the design is based on plant roots principles, the system described represents a useful platform for the experimental validation of theories and hypotheses concerning actual plant root systems.

## Results

### System Design

**Figure 3:**
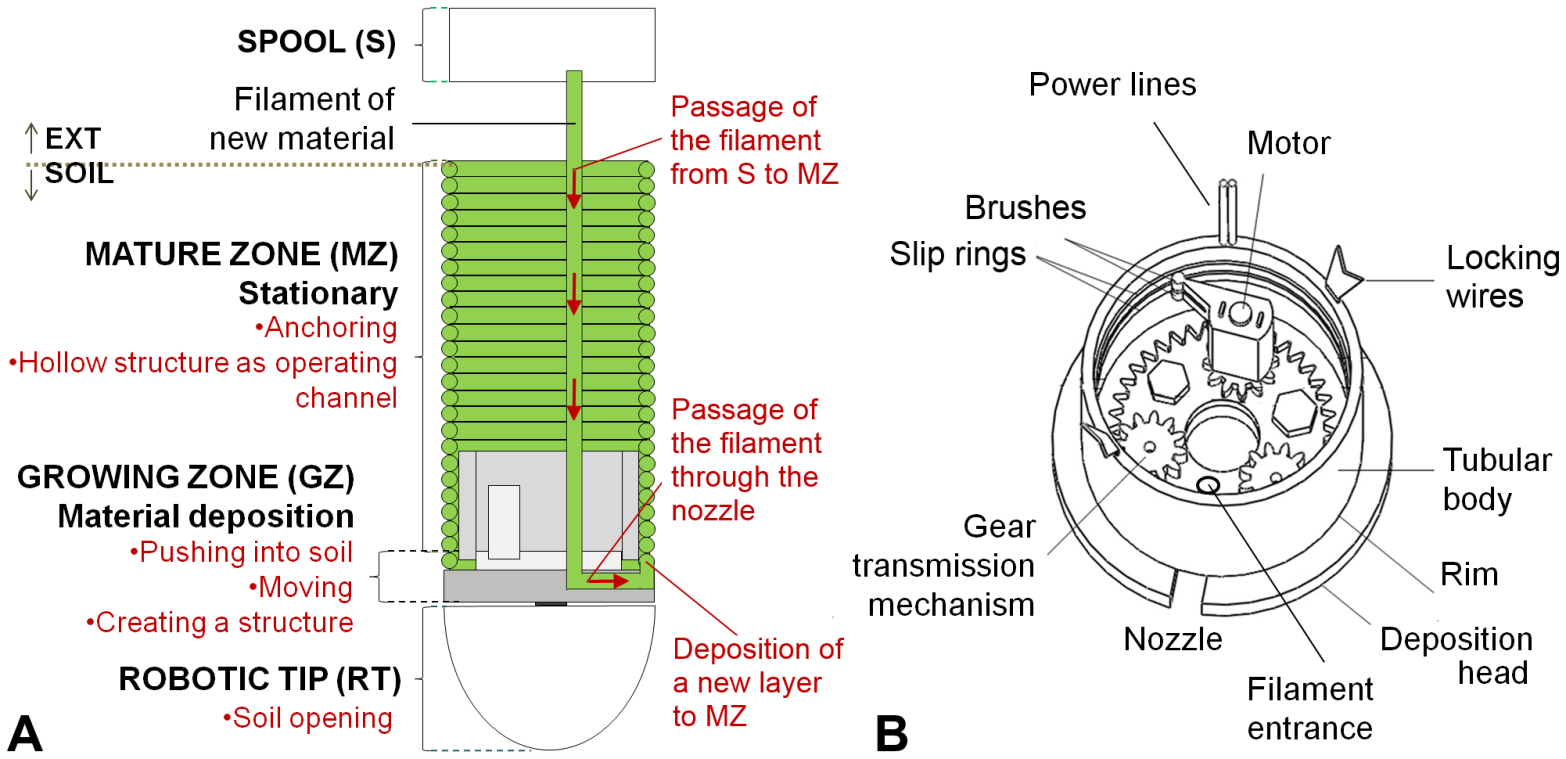
Robot Design. Figure (a) gives a schematic representation of the cross section of the root-like device that grows from the tip. The functional zones are the growing zone (GZ), the mature zone (MZ), the robotic tip (RT), and the spool (S) with the filament. The growing zone imitates the meristematic region in living plant roots; the GZ exerts an axial force on the robotic tip by adding new material. The mature zone is created by the layer-by-layer addition of material (the filament) and is stationary with respect to the soil. The hollow structure of the MZ allows the passage of the filament (new material) from the spool to the growing zone. Figure (b) shows the 3D design of the growing zone. The filament of new material passes through the filament entrance and the nozzle (located on the deposition head) to the external side of the tubular body between the rim and the previous layer of the mature zone. The rotation of the deposition head with respect to the tubular body, generated by the motor through the transmission mechanism, results in the deposition process that builds the mature zone. The motor receives power through brushes in contact with slip rings. During soil penetration, the growing zone slips inside the mature zone; any rotational movement between the two zones is suppressed by the locking wires.

The decrease in energy observed in the EFT penetration trials (see the “Materials and Methods” section) was used as a guideline in the design of our exploratory robotic device. A root-growth approach at the apical region results in an energy-efficient solution that addresses the penetration issue. Based on these premises, we propose a bio-inspired design of a root-like robotic system that grows at the apical area by the addition of new material. Figure 3a shows the conceptual design of this system. The device is composed of a growing zone and a stationary mature zone. The mature zone consists of a hollow, tubular structure that allows the transfer of new material from a spool (external to the robotic root) and power to the growing zone.

The growing zone is a customized additive layering mechanism that generates a force for penetration into the soil and consists of: a) a rotating deposition head, b) a guiding nozzle on the deposition head, and c) a motor and a transmission mechanism (i.e., a gearset) (Fig. 3b). The material, which is in the form of a filament, can be easily transferred from the external spool to the nozzle at the periphery of the deposition head. The “growing” capability at the tip region is achieved with an additive manufacturing technique similar to Fusion Deposition Modeling (FDM) [73]. The layer-by-layer deposition creates the mature zone, which uses the tubular body as a support structure. The rotation of the deposition head is driven by a motor coupled to the transmission mechanism. The tubular body moves axially inside the mature zone in a passive manner, and any rotational movement between them is prevented by locking wires installed on the body. The axial slipping movement induced by the addition of filament material allows the new material to be automatically distributed on the surface of the deposition head, in the growing zone (Fig. 3a). To prevent any twisting between the filament and the motor power cables, the power is transferred by two annular electrodes (i.e., slip rings) that are fixed to the internal surface of the tubular body and are always in contact with the brushes connected to the rotating motor. The cables are connected to the electrodes through an axial hole in the tubular body (Fig. 3b).

**Figure 4:**
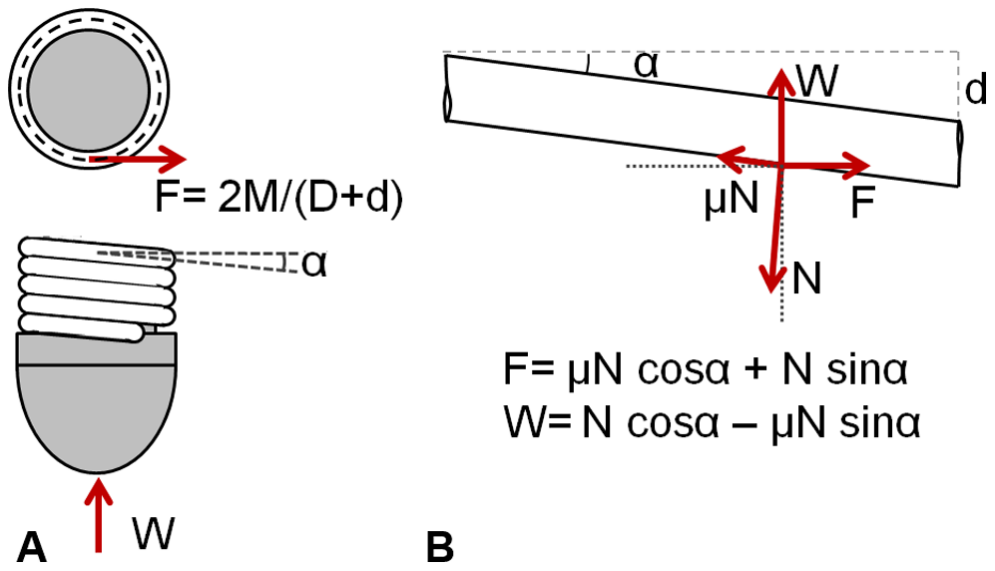
Force diagram for overcoming soil resistance. a) Schematic top view and side view of the growing mechanism. Red arrows represent forces acting on the system during the deposition process: F is the force applied by the motor and W is the vertical resistance during penetration, where M is the torque required for the deposition head (shown in Fig. 3b) to overcome W, d is the thickness of the filament and D is the external diameter of the tubular body b) Equilibrium of forces acting on the filament for one complete turn unwound, where α is the angle made by the helix of the deposited filament with respect to a plane perpendicular to the axis of the tubular body, N is the reaction force, and μ is the friction coefficient between filament and deposition head.

The rotary motion of the deposition head is converted into a linear motion at the tip that provides the motive force for overcoming soil resistance (Fig. 4a). If we assume that the diameter of the filament remains constant during the growth process, one deposition cycle of the tip penetrates by a distance equal to the filament diameter. The penetration depth, *P,* resulting from the deposition of the filament (with length *L* and diameter *d*) around the tubular body (with external diameter *D*) is given as:
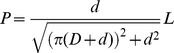
(1)


Similar to a screw, the force transmitted from the anchored mature zone to the advancing tip is achieved by exploiting the helical deposition pathway of the filament material. If the deposited filament material from one complete turn is unwound, the helix angle α (the angle made by the helix of the deposited material with a plane perpendicular to the axis of the tubular body) can be related to the geometrical dimensions of the system:
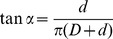
(2)


The two main sources of friction in the growing zone related to the passage and the deposition of the filament are the contact between the filament and the internal part of the nozzle and the contact between the filament and the rim of the deposition head, which can be considered a unique source of friction. The vertical resistance *W* is the result of soil resistance *W_s_* and frictional losses *W_f_* related to the slipping movement of the tubular body inside the mature zone. If we consider the equilibrium of the horizontal and vertical forces acting at a point on the newly deposited section of filament (Fig. 4b), then the torque *M* required by the deposition head to overcome the vertical resistance *W* caused by friction between the filament and the deposition head (with the coefficient of friction *μ*) is:
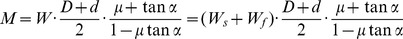
(3)


If a complete deposition cycle is considered and the vertical resistance is assumed to be locally constant (it increases for deeper layers), the efficiency of the deposition process is given by:
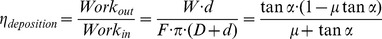
(4)


Thus, according to (2), the efficiency of the deposition mechanism depends on the diameter *D* of the tubular body, the diameter d of the material filament and the friction between the filament and the deposition head. The efficiency of the total actuation system (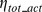) is the result not only of the deposition process but also of the performances of the motor (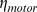) and of the transmission mechanism (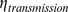) as follows:
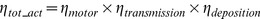
(5)


The rotational movement of the deposition nozzle creates torsion in the filament that can cause unwanted nodes or curves. The torsion is countervailed by a rotary movement of the external spool on which the filament is wound.

The helical deposition of the filament in our system has similarities with a screw-based power transmission mechanism. Because of the large load carrying capacity, screws have traditionally been used as a mechanism to convey removed materials in tunneling and drilling [2]. Moreover, screws have been exploited as self-tapping and drill-free penetration systems. For example, in [7], the penetration into the soil occurs because of a strong interaction between the helical thread and the substrate: the movement of the whole body with respect to the environment determines an increase in the energy needed for penetration and this increase becomes larger for deeper probe penetration. For the proposed device, the similarity with a screw is exclusively related to the conversion of a rotary motion to a linear motion and the transmission of power. By contrast, from the tip-soil interaction viewpoint, our device penetrates by soil compaction around the tip, and no mass is moving along the axis of the system (i.e., soil removal along the borehole). In our case, the penetration process benefits from the absence of peripheral friction along the mature zone.

### System Prototype

**Figure 5:**
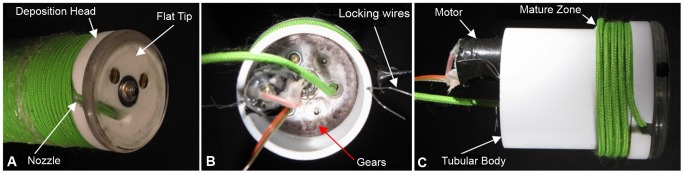
System Prototype. Figure (a) shows a picture of the root-like growing device. In the deposition head, a decoupled flat tip is assembled in order to eliminate any drilling effect in the penetration process. Figures (b) and (c) show the top and side views, respectively, of the device at the initiation of the mature zone. From the spool (not shown), the filament is drawn by the motor through the filament entrance into the nozzle. The locking wires are spring steel and prevent relative rotational movement between the tubular body and the mature zone.

Based on the design proposed in the previous section (Fig. 3), a prototype of the root-like robotic system was developed and tested. The robotic root can be used to imitate the growth zone in actual plant roots and validate its role in root growth. The material used to grow the system is a polypropylene (PP) filament (nominal diameter d = 2.5 mm). The deposition head (external diameter D_hs_ = 57 mm) and the tubular body (external diameter *D* = 50 mm) are constructed of polytetrafluoroethylene (PTFE) to decrease the frictional effects during the filament deposition. A groove (width = 5 mm, length = 27 mm, depth = 4 mm) in the deposition head acts as a nozzle for guiding the filament onto the outer surface of the tubular body (Fig. 5, Video S2). Through the rim (thickness = 3.5 mm) created by the deposition head and the tubular body on the tip of the system, the deposited filament creates the penetration action. The rotation of the deposition head is driven by a 2.37 W motor with a 246∶1 gear ratio (FAULHABER GMBH & CO.KG, Shonaich, Germany) coupled to a transmission mechanism containing a planetary gearset. The transmission mechanism consists of three spur gears that are mounted on a carrier disc and engage an internal gear (number of teeth for the internal gear = 37, number of teeth for the spur gears = 12). The gears and carrier were fabricated from Plexiglas using a laser cutting process (VersaLaser VLS3.50, Universal Laser Systems, Scottsdale, AZ). The internal gear is fixed to the bottom of the tubular body. The motor, which is mounted on the top of the carrier, turns one of the spur gears and determines the rotation of the carrier and the deposition head (Fig. 5b).

The locking wires are made from spring steel wire and mounted in the upper edge of the tubular body (Fig. 5b). The ends of these wires protrude into the mature zone and eliminate any relative rotational movements between the mature zone and the tubular body while allowing axial slipping.

**Figure 6:**
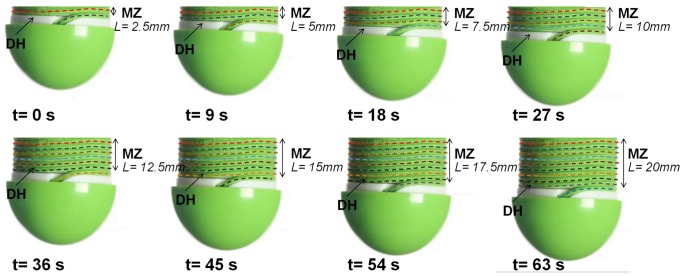
Deposition process and growth of the root-like device. The figure shows the sequence of the growth process. By rotating the deposition head (DH, in white), the filament passes through the nozzle and is deposited around the outer surface of the tubular body (shown in Figs. 3 and 5c). The penetration force is provided by the deposition of the filament at the growing zone (GZ), while the mature zone remains strongly anchored to the soil. The newly deposited filament is always located on the top of the DH, as shown by the colors.

Figure 6 shows the sequence of movements of the system as it grows.

### Validation

**Figure 7:**
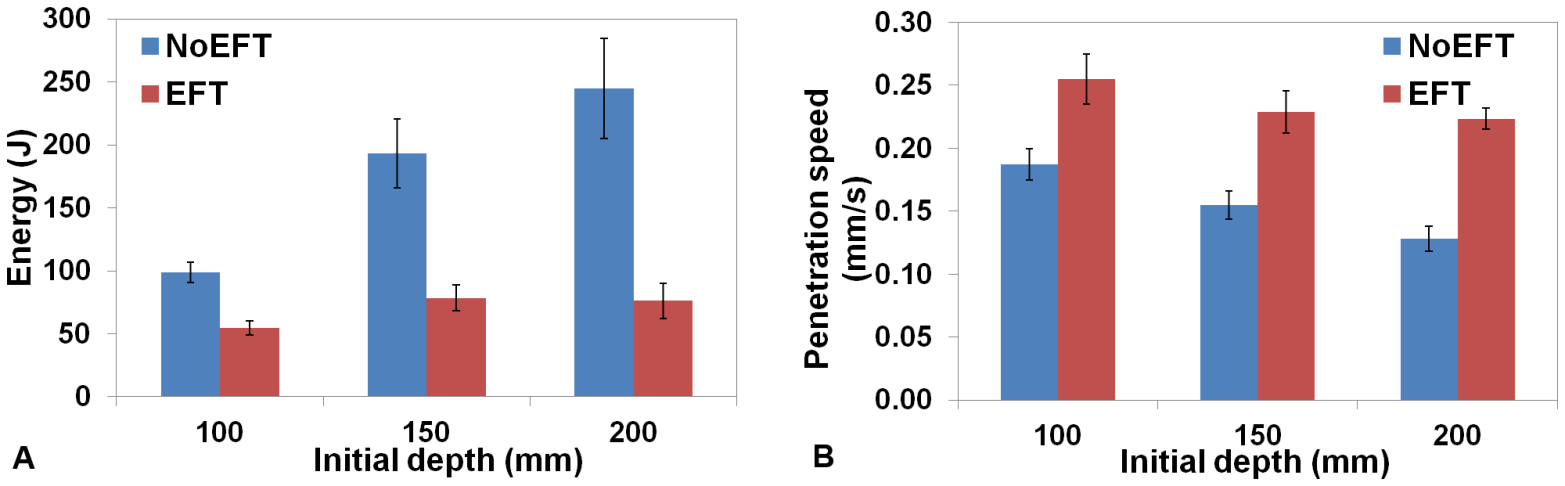
Energy consumption of the root-like device prototype in EFT and NoEFT penetration trials. Figure (a) shows the energy required for penetrating 30 mm of artificial soil (POM granules, diameter = 4 mm) in EFT (Red) and NoEFT trials (blue) at various initial depths (100, 150, 200 mm). For each initial depth, 5 tests were performed. Bars are the mean values over 5 repetition for each depth and condition (EFT and NoEFT). Error bars represent the standard error of the mean. Figure (b) shows the penetration speed achieved. The experimental conditions are described in the “Materials and Methods” section and shown in Fig. 8.

As reported in Materials and Methods, the prototype was tested in different depths and penetration conditions (EFT and NoEFT penetrations). Data are reported as means ± standard deviations (Fig. 7). For all the initial depths tested, the energy required for penetration was lower with EFT than with NoEFT (Fig. 7a). Because of the increasing soil pressure, the required energy increased with the initial depth. Notably, the increase in energy for the EFT penetrations had a gradual slope, from 54.7±5.5 J at a depth of 100 mm to 76.3±13.9 J at 200 mm, whereas for NoEFT, the results showed a rather constant and sharp slope, with energies between 98.8±7.8 J at 100 mm and 244.9±39.9 J at 200 mm. The reduction in energy consumption with EFT ranged from 45% at 100 mm depth to 70% at 200 mm depth. The standard deviations of the results, described by error bars in Fig. 7, increased with the initial depth.

Corresponding to greater depths, the penetration speed decreased in both types of penetration because of the higher mechanical impedance of the artificial soil. The EFT penetration speeds, averaged over the penetration time, were approximately 30–40% higher than the NoEFT speeds (Fig. 7b).

## Discussion

The ability of roots to penetrate soil appears to be mainly attributable to growth at the apical region. In fact, if penetration of the root into the soil resulted from a force caused by growth of the root in the region proximal to the stem, the root would have to generate higher penetration forces because of the increased friction on the flanks. Furthermore, the deformation of the root structure due to the buckling and mechanical bending would compromise the root steering capabilities essential for the exploration. From a biological perspective, a hypothetical root that grows from the stem would be subject to a higher risk of tissue damage caused by both friction and buckling. Thus, root growth from the tip is a beneficial adaptation to the soil environment.

Although growth is essential for the optimal interaction of the root with the soil, its contribution to soil penetration is difficult to quantify in living roots. To the best of our knowledge, studies concerning penetration capabilities have mainly addressed the rheological properties of the apex and its role in friction reduction [53], [55], morphological changes [43], [54], [74], and root penetration pressure [49], [66], [75]. In the proposed biomimetic approach, the root-like growing device represents a translation (simplification) of biological concepts into an engineering system for soil penetration and exploration. This artificial growing system is able to create its own structure with a customized deposition technique. Similar to the probes commonly used for soil exploration, such as penetrometers [76], [77], our device is able to create and follow a path into the soil without deviation or buckling: the self-developed structure anchors into the soil and provides a thrust force localized at the tip level. This device could provide a penetration resistance comparable to that experienced by growing roots. Moreover, the total thrust force can be used to surmount axial soil resistance without being dissipated by lateral friction: similar to living roots, the growing device succeeds in efficiently penetrating a granular medium. The experimental trials comparing EFT and NoEFT penetration demonstrated that the device penetrates soil faster and with less energy consumption using EFT penetration. We found lower energy consumption (from 45% to 70%) using EFT compared with NoEFT for initial depths ranging between 100 mm and 200 mm. This characteristic, obtained by the growing approach, is the key feature of our device, which is intended to perform an autonomous and nondestructive exploration of the shallow layers of soil.

In a previous study [45], we demonstrated the effect and contribution of the apex morphology in plant root soil penetration. The root-like device in the present study does not currently include a tip with an optimal shape for penetrating a granular medium, nor is it equipped with a sloughing mechanism such as that described in [72]. Therefore, the efficiency of this growing system can be increased by adding these two features. If supported by the growing mechanism presented here, a sloughing tip would burrow into soil with no peripheral friction because of the stationary interaction of the mature zone with the peripheral soil. Moreover, by depositing the filament asymmetrically around the outer surface of the tubular body, bending can be achieved, which would enhance the exploration capabilities of the growing robot.

The proposed system could be used as an autonomous tunneling system, especially for granular substrates (e.g., deserts, regolith, or debris after natural disasters), or for space applications.

This work is based on a breakthrough concept of imitating the growth process in robots. Up to now, the concepts of reconfiguration have been developed in the self-reconfigurable robots, in which pre-existing/predefined modules are used to flexibly build and vary their structure [78]–[81]. These discrete modules can attach to each other by means of magnetic [82], mechanical [83], and passive chemical [84] bonding connectors. This powerful idea allows the system to adapt to its environment and is highly versatile for task orientation. Some unconventional materials and techniques have been used to progress beyond the lattice-type structure of conventional reconfigurable robots. In [85], the modules are linked and attached together with a second robot that injects foam to construct the desired structure. In [86], the concept of ‘robotic body extension’ was demonstrated by using an additive layering technique based on thermoplastic adhesives (TPAs). Unlike the conventional rapid prototyping processes, the structures fabricated in the present study can be reshaped and reconfigured by controlling a physical parameter, such as temperature. The flexibility provided by other additive manufacturing techniques, including shape deposition manufacturing (SDM) [87] and smart composite microstructure (SCM) [88], have also been extensively used to simultaneously fabricate and assemble the components of devices in an integrated manner. By contrast with the previous research, the proposed root-like device grows through a monotonic process that continuously adds new material. The technological approach used to grow the mature body is also responsible for the penetration function because the robot is pushing the soil while it is growing. Thus, the creation process has not only a functional meaning but also a structural one, and both of these features are implemented simultaneously on board the robotic system itself. The concept of a growing robot may spawn new approaches in assembly and robotics by incorporating the manufacturing technology inside the robot. Self-reconfigurable structures could use the growth process to construct themselves directly on the site of interest.

Being designed on the basis of biological functions, the root-like growing robot and its future developments can be applied as tools to validate hypotheses on plant growth that may be difficult to demonstrate *in vivo*. The full potential of biomimetic robotics remains to be explored and offers interesting and demanding research challenges.

## Materials and Methods

### Experimental Setup for Soil Penetration

The prototype with a flat tip was tested in a cohesionless granular medium consisting of polyoxymethylene (Ultraform N2320 003, BASF, Ludwigshafen, Germany) plastic beads (diameter = 4 mm). Repeatable results can be obtained with this substrate because of its low moisture absorption (0.2% under experimental conditions of T = 23°C and 50% relative humidity), favorable mechanical properties and wear resistance properties [89]. The artificial soil was kept in a cylindrical container (height = 250 mm, diameter = 200 mm) at room temperature (T = 21±1.5°C) and had a bulk density of 870 g/l and a void ratio of 0.61.

Under these conditions, by using a 30° circular stainless steel cone (base area 323 mm^2^ and 20.27 mm diameter) with a drive shaft, as defined by ASAE S313.3 [13], the artificial soil strength was characterized by measuring the resistance of the soil to penetration by a cone driven at a constant rate [90], [91]. A resulting cone index (i.e., the force of insertion per unit cone base area, with the number of repetitions = 20 and the standard deviation <10%) of 0.017, 0.033, or 0.059 MPa at 100, 150, or 200 mm depths, respectively, was measured according to the standard ASAE EP542 procedure [92].

**Figure 8:**
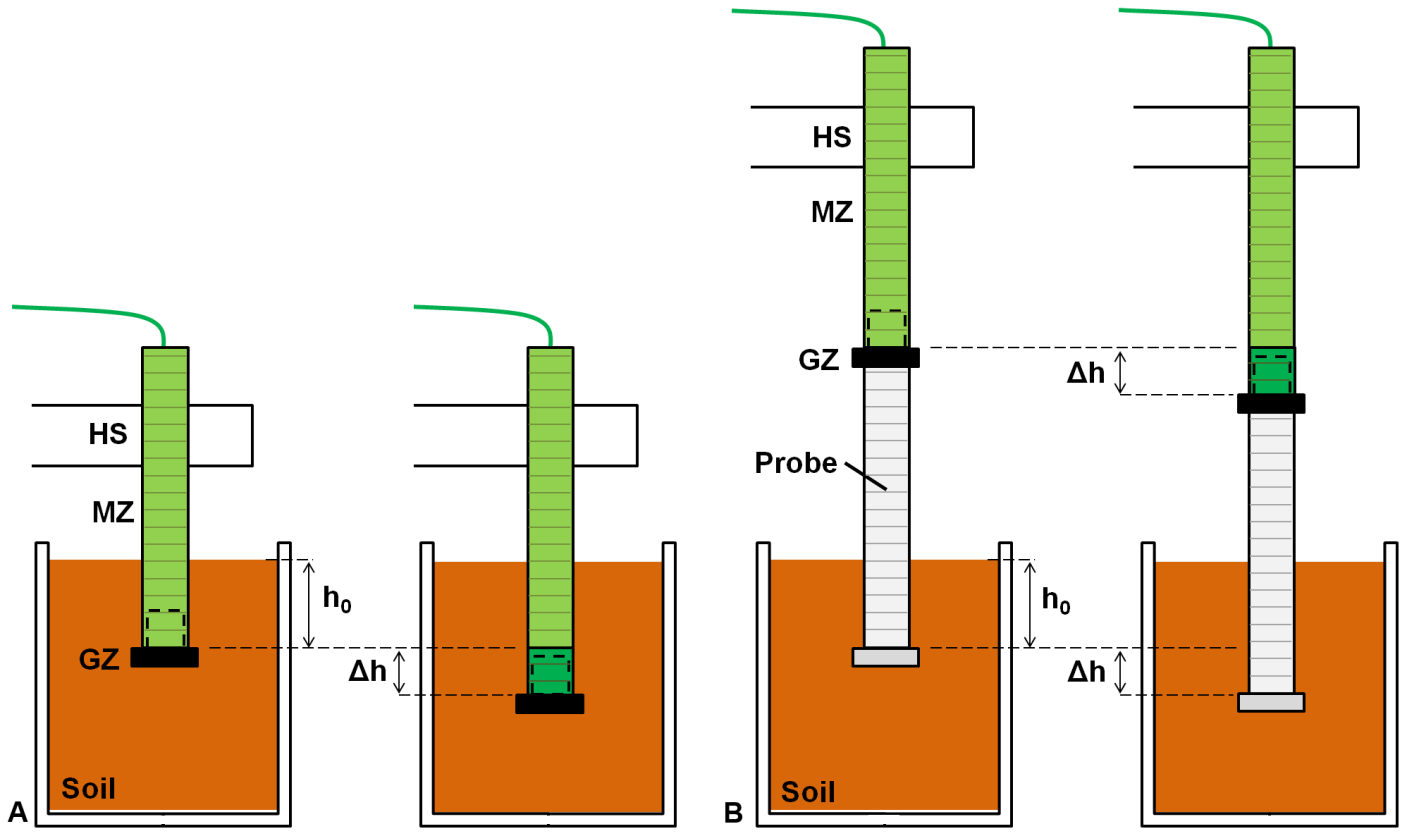
Schematic of the experimental setup for soil penetration with and without elongation from the tip (EFT and NoEFT, respectively). Figure (a) shows the setup for the evaluation of the energy required in the EFT tests, at the beginning (on the left) and at the end (on the right) of a soil penetration trial. In the initial position, the device is positioned in the soil. The growing zone (GZ) is placed at depth h_0_ (h_0_ = 100, 150, 200 mm), and the mature zone (MZ) is fixed to a holding structure (HS). The GZ adds new material at the tip, thus increasing the MZ length until it penetrates a total of Δh (30 mm). Figure (b) shows the setup for the evaluation of the energy required in the NoEFT tests, at the beginning (on the left) and at the end (on the right) of a soil penetration trial. In the initial position, a probe (F, light grey) with the same dimensions and geometric properties as the prototype is positioned at depth h_0_ in the soil (h_0_ = 100, 150, 200 mm). The prototype is attached to the HS and pushes the probe from the top. The GZ generated the vertical (penetration) force in both the EFT and NoEFT trials in order to have comparable results.

A dedicated setup was developed to perform the EFT and NoEFT penetration trials. This approach guaranteed that the effects of parameters such as manufacturing, materials, and components were equal in both experimental cases. In the EFT penetration tests, the mature zone (MZ) of the device was fixed to the holding structure (HS, Fig. 8a, Video S3), and the growing zone (GZ) was positioned in the artificial soil at a prescribed initial depth (h_0_). In the NoEFT penetration tests (Fig. 8b, Video S3), the entire growing system (MZ+GZ) was fixed externally to the soil, and another probe (Fig. 8b) was added to the growing tip. This new probe and tip were geometrically equal to the growing system (MZ+GZ) used in the EFT penetration tests. The probe was inserted into the soil with the tip at the prescribed initial depth h_0_ and was driven by the external growing system.

### Energy Estimation in EFT and NoEFT Trials

The energy consumption of the penetration probe was calculated in both the EFT and NoEFT trials. In the former, the device penetrated axially by growing from the tip while the peripheral body of the device remained stationary relative to the soil. In the latter, a probe with same shape and dimensions as the device was pressed from the top; i.e., the entire body of the probe moved relative to the soil. For both the cases, the penetration tips were inserted into the soil at an initial depth h0 (100, 150 and 200 mm), and in each experiment the energy required to penetrate 30 mm soil was measured. To calculate the energy consumed, a precision power source/measurement unit (Agilent B2912A) with the capability to control and measure both voltage and current was used to measure the motor current and the time while the voltage was held constant (V = 4 V); the data were recorded on a PC. For each initial depth, 5 tests were performed; the mean values of energy consumed and standard deviations were calculated. The soil was redistributed after each penetration trial to restore the initial packing state of the grains (parameterized by bulk density and void ratio [93], [94], which was locally changed because of the tip advancement and the consequent soil compaction during penetration.

## Supporting Information

Video S1 **Schematic animation of elongation from the tip (EFT) process.**
(MP4)

Video S2 **System Prototype.** The sequences are speeded up 2X.
(MP4)

Video S3 **Experimental Setup for Soil Penetration with and without elongation from the tip (NoEFT).** The sequences are speeded up 8X.
(MP4)
